# The VP1 unique region of human parvovirus B19 and human bocavirus induce lung injury in naïve Balb/c mice

**DOI:** 10.1371/journal.pone.0202667

**Published:** 2018-08-16

**Authors:** Chun-Yu Lin, Yu-Han Chung, Ya-Fang Shi, Bor-Show Tzang, Tsai-Ching Hsu

**Affiliations:** 1 Division of Allergy-Immunology-Rheumatology, Department of Internal Medicine, Chi-Mei Medical Center, Tainan, Taiwan; 2 Department of Internal Medicine, National Cheng Kung University Hospital, College of Medicine, National Cheng Kung University, Tainan, Taiwan; 3 Institute of Biochemistry, Microbiology and Immunology, Chung Shan Medical University, Taichung, Taiwan; 4 Department of Biochemistry, School of Medicine, Chung Shan Medical University, Taichung, Taiwan; 5 Immunology Research Center, Chung Shan Medical University, Taichung, Taiwan; 6 Clinical Laboratory, Chung Shan Medical University Hospital, Taichung, Taiwan; University of Kansas Medical Center, UNITED STATES

## Abstract

Both human parvovirus B19 (B19V) and human bocavirus (HBoV) are known to be important human pathogens of the *Parvoviridae* family. Our earlier investigation demonstrated that both B19V-VP1u and HBoV-VP1u have a significantly disruptive effect on tight junctions (TJs) in A549 cells, implying the essential role of parvovirus in airway infection and lung injury. However, no direct evidence that B19V-VP1u and HBoV-VP1u induce lung injury exists. The present study further investigates the induction of lung injury by B19V-VP1u and HBoV-VP1u in naïve Balb/c mice following subcutaneous injection of PBS, recombinant B19V-VP1u or HBoV-VP1u. The experimental results reveal significantly increased activity, protein expression and ratio of matrix metalloproteinase-9 (MMP-9) to MMP-2 in Balb/c mice that received B19V-VP1u or HBoV-VP1u compared to those that received PBS. Significantly higher levels of inflammatory cytokines, including IL-6 and IL-1β, and greater lymphocyte infiltration in lung tissue sections were detected in mice that received B19V-VP1u or HBoV-VP1u. Additionally, significantly increased levels of phosphorylated p65 (NF-κB) and MAPK signaling proteins were observed in lung tissue of mice that received B19V-VP1u or HBoV-VP1u compared to those of mice that received PBS. These findings demonstrate for the first time that B19V-VP1u and HBoV-VP1u proteins induce lung inflammatory reactions through p65 (NF-κB) and MAPK signaling.

## Introduction

A growing body of evidence has implicated viral respiratory tract infection a predominant risk factor associated with a variety of lung illnesses, such as chronic obstructive pulmonary disease (COPD), asthma and cystic fibrosis (CF) [1]. Infection with viruses, such as rhinovirus, influenza and respiratory syncytial virus (RSV), has been indicated as an important contributor to COPD exacerbations in 40% to 60% of all COPD patients [2]. Other longitudinal investigations have reported that episodes of rhinovirus- or RSV-infection resemble manifestations of asthma, especially in younger groups, and are related to the later development of asthma [3–5]. RSV infection accounts for up to one-third of hospitalizations, respiratory failure, and the chronic supplemental oxygen requirement of infants with cystic fibrosis (CF). Similar results have been obtained for children with CF who have impaired lung function [6–9].

The human parvovirus B19V (B19V) and human bocavirus (HBoV) are members of the *Parvoviridae* family known to be two important parvoviruses that are responsible for many human diseases [10–13]. The VP1 unique region (VP1u) of both B19V and HBoV has the motif and activity of secreted phospholipidase (sPLA2) which has been demonstrated to have an essential role in infectivity and induction of inflammation in host cells [14–16]. Notably, B19V and HBoV are also respiratory viruses, which can be transmitted through respiratory tract infection and are strongly associated with the development of various respiratory diseases such as sinusitis, pharyngitis, obstructive bronchitis, bronchopneumonia and asthma [17–21]. These findings reveal that parvoviruses have a critical role in airway infection and the development of lung disease.

Although previous studies have suggested a connection between parvovirus infection and lung disease [22–23], no direct evidence of this connection has been reported. Our recent study revealed that HBoV-VP1u and B19V-VP1u have a significantly disruptive effect on tight junctions (TJs) in A549 lung cells [24]. Accordingly, the current study further investigates the induction of lung injury by B19V-VP1u and HBoV-VP1u in naïve mice following subcutaneous injection of recombinant B19V-VP1u and HBoV-VP1u proteins, with the goal of clarifying the potential involvement of human parvoviruses in the induction of lung disease.

## Materials and methods

### Preparation of recombinant human B19V-VP1u, HBoV-VP1u, B19V-VP1uD175A and HBoV-VP1uL48R proteins

DNA fragments encompassing B19V-VP1u, B19V-VP1uD175A [25], HBoV-VP1u [24] (TW125_07: GeneBank accession number EU984241.1, provided by Centers for Disease Control, Taipei, Taiwan) and HBoV-VP1uL48R [26] were amplified by polymerase chain reaction (PCR). The DNA fragments of B19V-VP1u, B19V-VP1uD175A, HBoV-VP1u and HBoV-VP1uL48R were then separately ligated into pET-32a vector (Novagene, Cambridge, MA). The products of ligations, pET32a-B19V-VP1u, pET32a-B19V-VP1uD175A, pET32a-HBoV-VP1u and pET32a-HBoV-VP1uL48R, were then transformed into Escherichia coli BL21-DE3 competent cells (Invitrogen, Carlsbad, CA). The B19V-VP1u, B19V-VP1uD175A, HBoV-VP1u and HBoV-VP1uL48R recombinant proteins were expressed by inducing with IPTG (1 mM) and purified with a Ni-NTA spin column (Qiagen, Chatsworth, CA, USA) and PureProteome™ Nickel Magnetic Beads (EMD Millipore, CA, USA). The bvPLA2 was used as positive control that exhibits the PLA2 catalytic activity of 0.430±0.016 μmol/min/mL. Two μg B19V-VP1u and HBoV-VP1u recombinant proteins were analyzed to measure their sPLA2 activity that exhibits 0.386±0.015 μmol/min/mL and 0.564±0.032 μmol/min/mL, respectively. No sPLA2 activity was detected in recombinant B19V-VP1uD175A and HBoV-VP1uL48R proteins.

### Ethics and animals

Fifteen female BALB/c mice aged 6 weeks were purchased from the National Laboratory Animal Center, Taiwan. Approval for this study was obtained from the Institutional Animal Care and Use Committee of Chung Shan Medical University, Taichung, Taiwan (No. 1241). The animals were kept under a 12-h light-dark cycle and ambient temperature was maintained at 25°C. Animals were allowed free access to water and standard laboratory chow (Lab Diet 5001; PMI Nutrition International Inc., Brentwood, MO, USA). At the age of 8 weeks, all mice were randomly divided into three groups (five mice per group) and injected subcutaneously with 20 μg purified B19V-VP1u or HBoV-VP1u recombinant proteins or phosphate-buffered saline (PBS) mixed 1:1 (v/v) with Freund's complete adjuvant (Sigma-Aldrich, UK). The mice were boosted with 10 μg B19V-VP1u and HBoV-VP1u recombinant proteins or phosphate-buffered saline (PBS) mixed 1:1 (v/v) with Freund's incomplete adjuvant (Sigma-Aldrich, UK) every two weeks for 3 times and then sacrificed at the age of 16 weeks by CO_2_ asphyxiation. The heart blood and lung tissues were collected and stored at −80°C until use.

### Hematoxylin-eosin staining

The lung samples of animals were excised and soaked in formalin and covered with wax. The section slides were prepared by deparaffinization and dehydration and were passed through a series of graded alcohols (100%, 95% and 75%) for 15 min each. The slides were then dyed with hematoxylin. After gently rinsing with water, each slide was then soaked with 85% alcohol, 100% alcohol I and II for 15 min each. Finally, they were soaked with Xylene I and Xylene II. Photomicrographs were obtained using Zeiss Axiophot microscopes. For quantification, the number of infiltrated lymphocytes was counted in 4 randomly selected fields of a section slide. All measurements were made using at least 3 section slides from 3 independent animals.

### Preparation of tissue extract and immunoblotting

All procedures were performed at 4°C in a cold room. The lung tissue samples obtained from Balb/c mice were homogenized in 600 μl PRO-PREP™ solution (iNtRON Biotech, Korea) by 30 strokes using a Dounce Homogenizer (Knotes Glass, Vineland, NJ). The homogenates were centrifuged at 13,000 rpm for 10 min at 4°C, and the supernatant was then stored at −80°C until use. Protein concentration of tissue extracts was determined using bovine serum albumin as a standard and immunoblotting was performed as described previously [24]. Briefly, equal amount of protein samples (25μg per sample) were separated in a 10% or 12.5% SDS-PAGE and electrophoretically transferred to nitrocellulose (NC) membrane (Amersham Biosciences, Piscataway, NJ, USA). After blocking with 5% non-fat dry milk in PBS, antibodies against MMP9, MMP2, IL-6, IL-1β, NF-κB (p65), p-P38, p-JNK, p-ERK, (Santa Cruz Biotechnology, CA, USA) and β-actin (MAB1501, Chemicon; EMD Millipore, Temecula, CA, USA) were diluted in PBS with 2.5% BSA and incubated with NC membrane for 1.5h. The membranes were then washed twice with PBS-Tween for 1 h and reacted with secondary antibody conjugated with horseradish peroxidase (HRP) (Santa Cruz Biotechnology, Santa Cruz, CA, USA). Immobilon Western HRP Chemiluminescent Substrate (EMD Millipore) was used to detect the antigen-antibody complexes. The blots were scanned and quantified by a densitometry apparatus (Alpha-Imager 2200; ProteinSimple, San Jose, CA, USA).

### Gel zymography

The activities of matrix metalloproteinase-2 (MMP2) and matrix metalloproteinase-9 (MMP9) were analyzed by gelatin zymography assays as described previously [26]. Briefly, 25 μg lung tissue lysates from Balb/c mice were separated on a 12% sodium dodecyl sulfate-polyacrylamide gel electrophoresis (SDS-PAGE) gel containing 0.1% gelatin. The gels were washed for 30 min in 2.5% Triton X-100 and then soaked in the reaction buffer (40 mM Tris–HCl, pH 8.0, 10 mM CaCl_2_ and 0.02% NaN_3_) for 16 h. Gelatinolytic activity was visualized by staining the gels with 0.5% Coomassie brilliant blue R-250, destained with methanol–acetic acid water. Relative MMP levels were quantified by a gel documentation and analysis system (Alpha-Imager 2200; ProteinSimple, San Jose, CA, USA).

### Statistical analysis

All statistical analyses were performed using GraphPad Prism 5 software (GraphPad Software, CA) by one-way analysis of variance (One-way ANOVA) followed by Tukey multiple-comparisons test. Data were represented as the mean ± SEM and verified by at least three independent experiments. A value of P < 0.05 was considered statistically significant. Significant differences are indicated with a star symbol, as shown in the figures.

## Results

### B19V-VP1u and HBoV-VP1u proteins increase activity and expression of MMP-9 and MMP-2 in lung tissue of Balb/c mice

**Fig 1:**
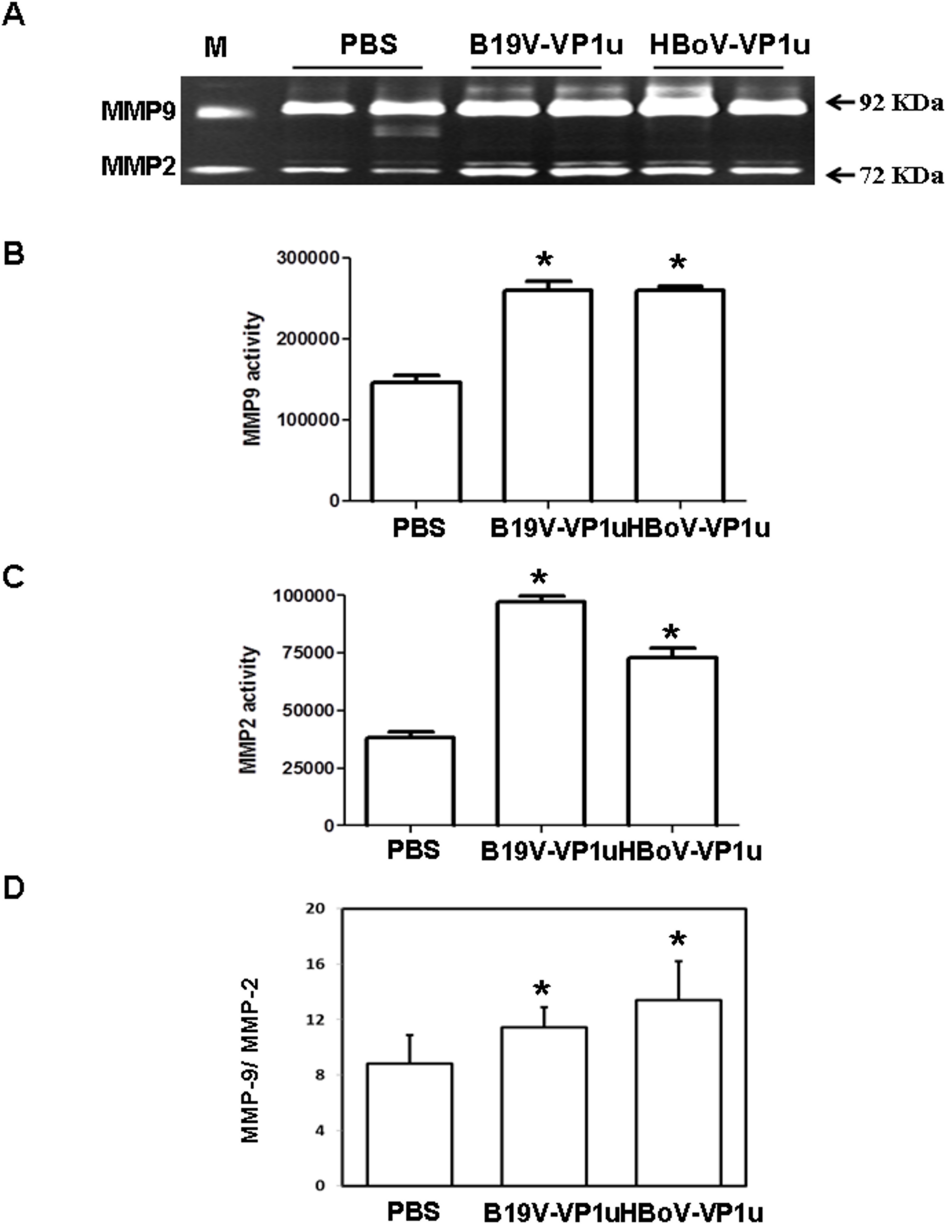
Activity of MMP-9 and MMP-2 in lung tissue of Balb/c mice. Activity of (A) MMP-9 and MMP-2 in lung tissue of Balb/c mice that received PBS, B19V-VP1u or HBoV-VP1u protein. Quantified results of (B) MMP-9, (C) MMP-2 and (D) ratio of MMP-9 to MMP-2. M is human serum and indicated as positive control. Similar results were observed in three repeated experiments, and * indicates a significant difference, P<0.05.

**Fig 2:**
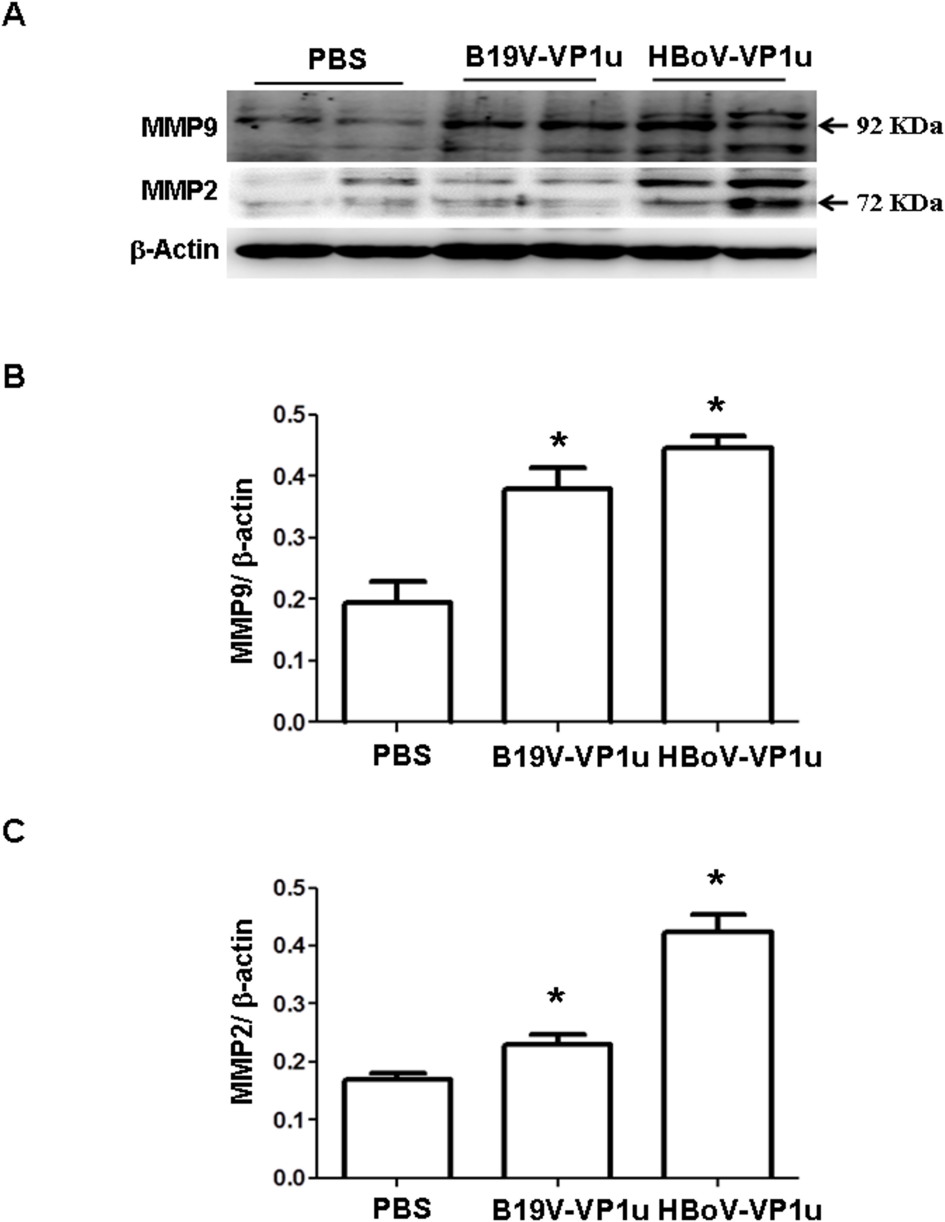
Expression of MMP9 and MMP2 proteins in lung tissue of Balb/c mice. Expression of (A) MMP-9 and MMP-2 proteins in lung tissue of Balb/c mice that received PBS, B19V-VP1u or HBoV-VP1u protein. Bars represent the relative protein amounts of (B) MMP9 and (C) MMP2 on the basis of β-actin. M is human serum and indicated as positive control. Similar results were observed in three repeated experiments, and * indicates a significant difference, P<0.05.

Elevated MMP-9 and MMP-2 activities have been linked to numerous lung disorders. To determine whether B19V-VP1u and HBoV-VP1u proteins cause lung injury in Balb/c mice, the activities and expressions of MMP-9 and MMP-2 were detected. Significantly increased activities of MMP-9 and MMP-2 were observed in lung tissue from Balb/c mice that received B19V-VP1u or HBoV-VP1u protein than in those mice that received PBS (Fig 1A). Fig 1B and 1C display quantitative results concerning MMP-9 and MMP-2 activities. Fig 1D displays the activity ratio of MMP-9 to MMP-2. The levels of MMP-9 and MMP-2 proteins were examined by immunoblotting. Significantly increased levels of MMP-9 and MMP-2 proteins were detected in lung tissue from Balb/c mice that received B19V-VP1u or HBoV-VP1u than in that of mice that received PBS (Fig 2A). Fig 2B and 2C display ratios of MMP-9 and MMP-2 to β-actin, respectively.

### B19V-VP1u and HBoV-VP1u proteins increase IL-1β and IL-6 expressions and lymphocyte infiltration in lung tissue of Balb/c mice

**Fig 3:**
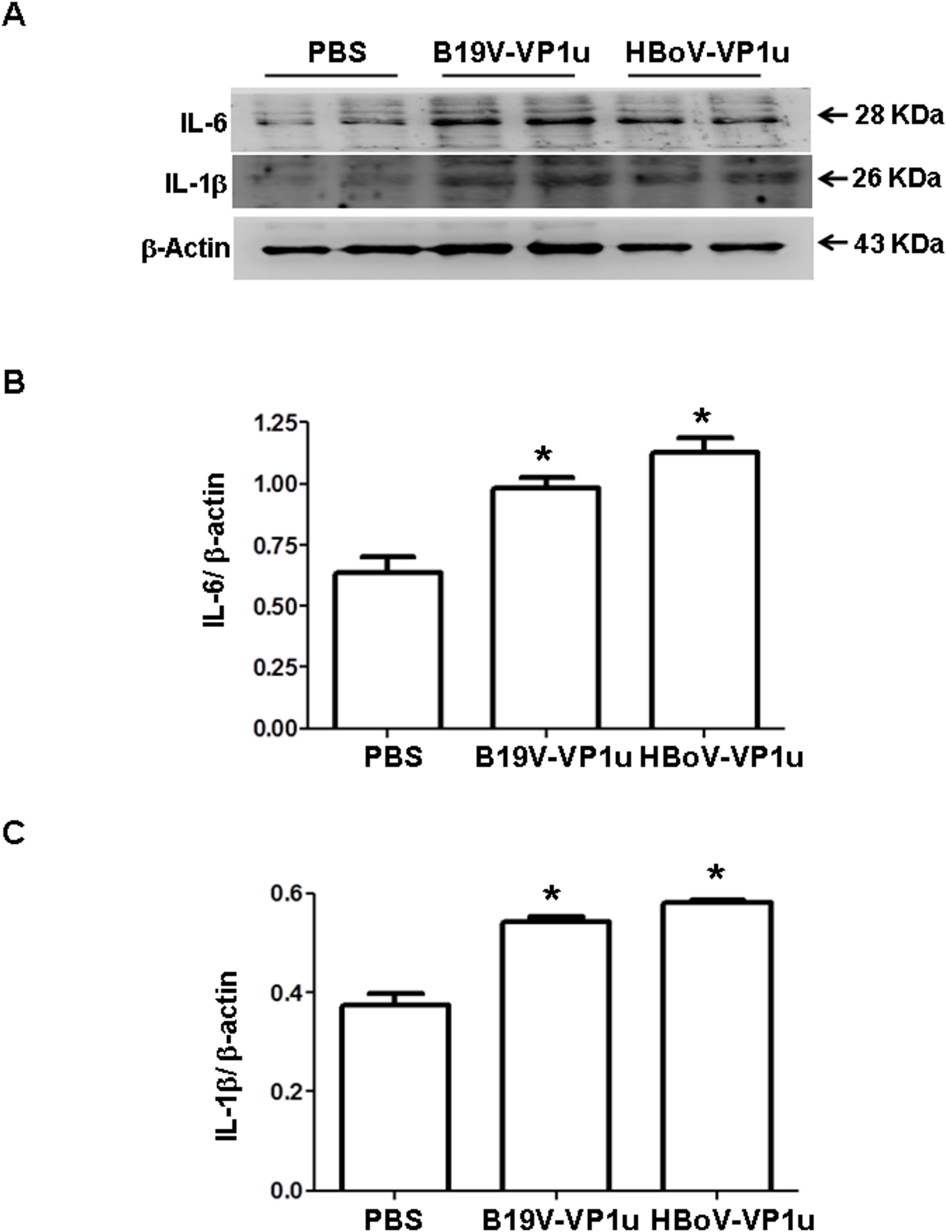
Expression of cytokine proteins in lung tissue of Balb/c mice. Expression of (A) IL-6 and IL-1β proteins in the lung tissue of Balb/c mice that received PBS, B19V-VP1u or HBoV-VP1u protein. Bars represent the relative protein amounts of (B) IL-6 and (C) IL-1β on the basis of β-actin. Similar results were observed in three repeated experiments, and * indicates a significant difference, P<0.05.

**Fig 4:**
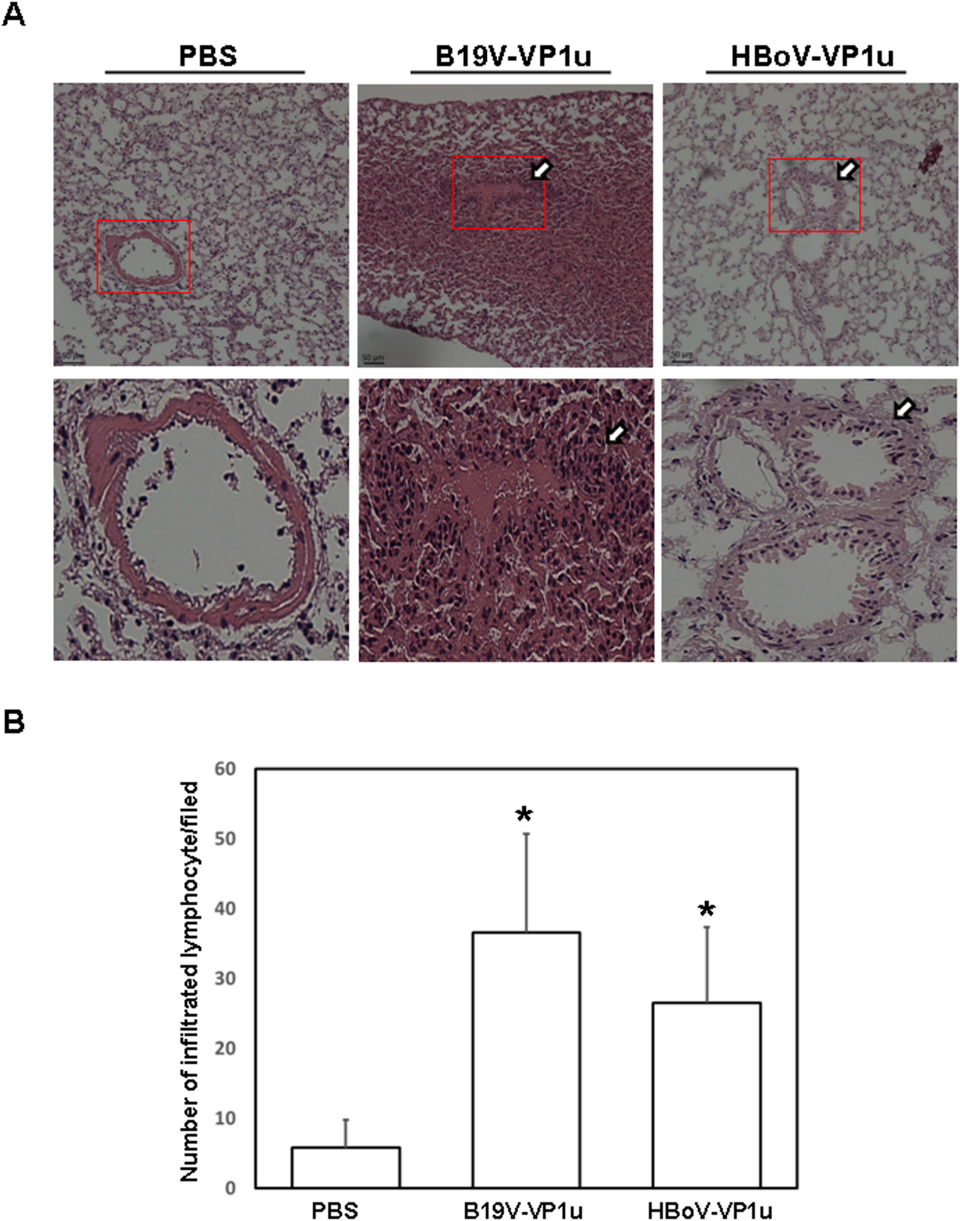
Lymphocyte infiltration in lung tissue of Balb/c mice. Lung tissues from Balb/c mice that received PBS, B19V-VP1u or HBoV-VP1u protein were obtained and (A) sections of the lung tissue were stained with hematoxylin eosin. The images of lung tissue sections were magnified by 200 times. Amplified images are shown in the lower panel of each section. Lymphocyte infiltration is indicated by an arrow. (B) The number of infiltrated lymphocytes was calculated as described in the section of materials and methods. * indicates a significant difference, P<0.05.

To investigate further whether B19V-VP1u and HBoV-VP1u cause lung injury in Balb/c mice, various inflammatory associated proteins, such as IL-1β and IL-6, were also examined. Significantly higher levels of IL-1β and IL-6 proteins were detected in lung tissue from Balb/c mice that received B19V-VP1u or HBoV-VP1u protein than in that from mice that received PBS (Fig 3A). Fig 3B and 3C show quantitative results concerning IL-1β and IL-6 proteins levels, respectively, relative to β-actin level. Hematoxylin and eosin staining was conducted to observe histopathological phenomena in lung tissue of Balb/c mice. Markedly greater lymphocyte infiltration was observed in lung tissue from Balb/c mice that received B19V-VP1u or HBoV-VP1u protein than in that from mice that received PBS (Fig 4). Fig 4B shows quantitative results.

### Expression of NF-kB (p65), p-ERK, p-JNK and p-P38 in lung tissue of Balb/c mice received B19V-VP1u or HBoV-VP1u protein

**Fig 5:**
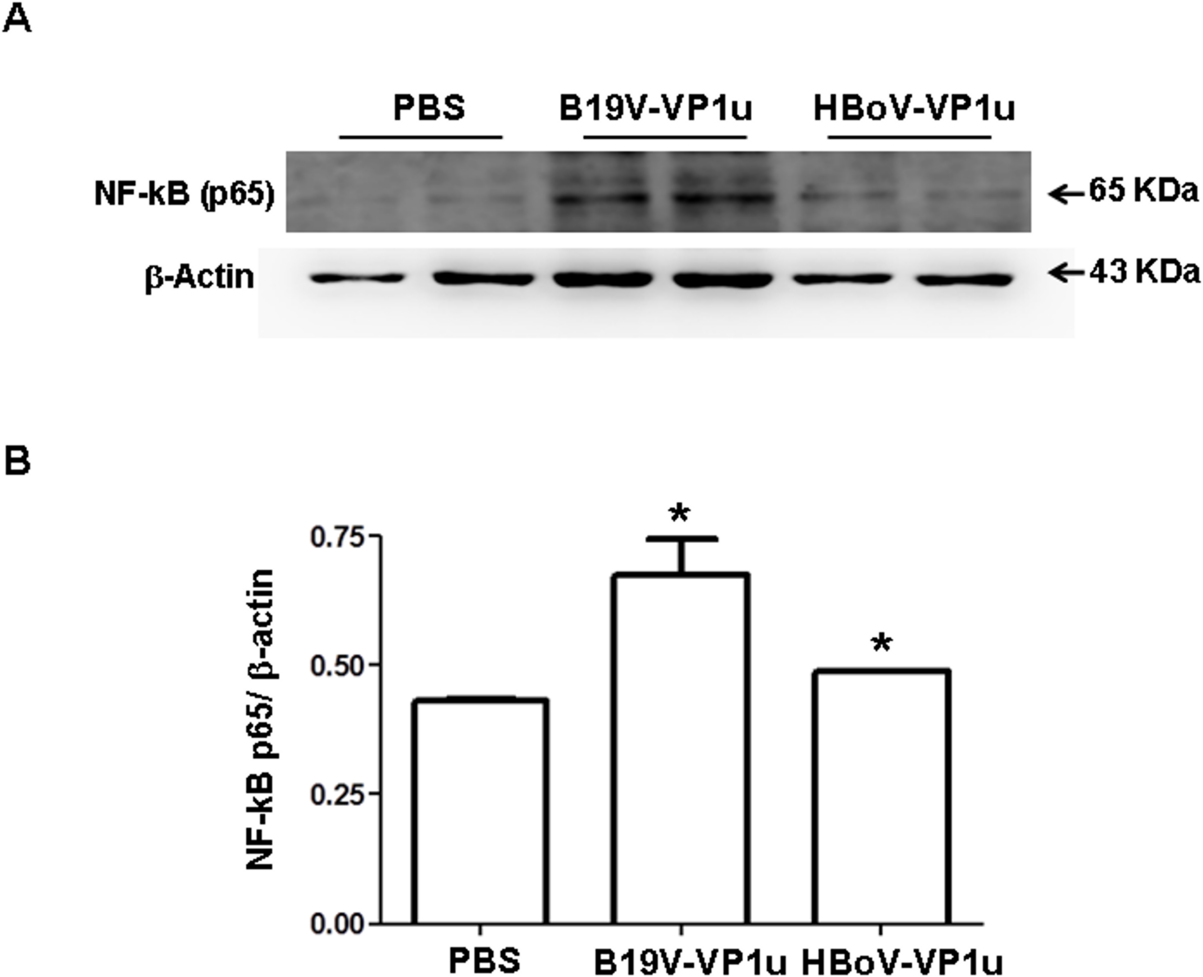
Expression of NF-kB (p65) protein in lung tissue of Balb/c mice. Expression of NF-kB (p65) protein in lung tissue of Balb/c mice that received PBS, B19V-VP1u or HBoV-VP1u protein. Bars represent the relative protein amounts of (B) NF-kB (p65) on the basis of β-actin. Similar results were observed in three repeated experiments, and * indicates a significant difference, P<0.05.

**Fig 6:**
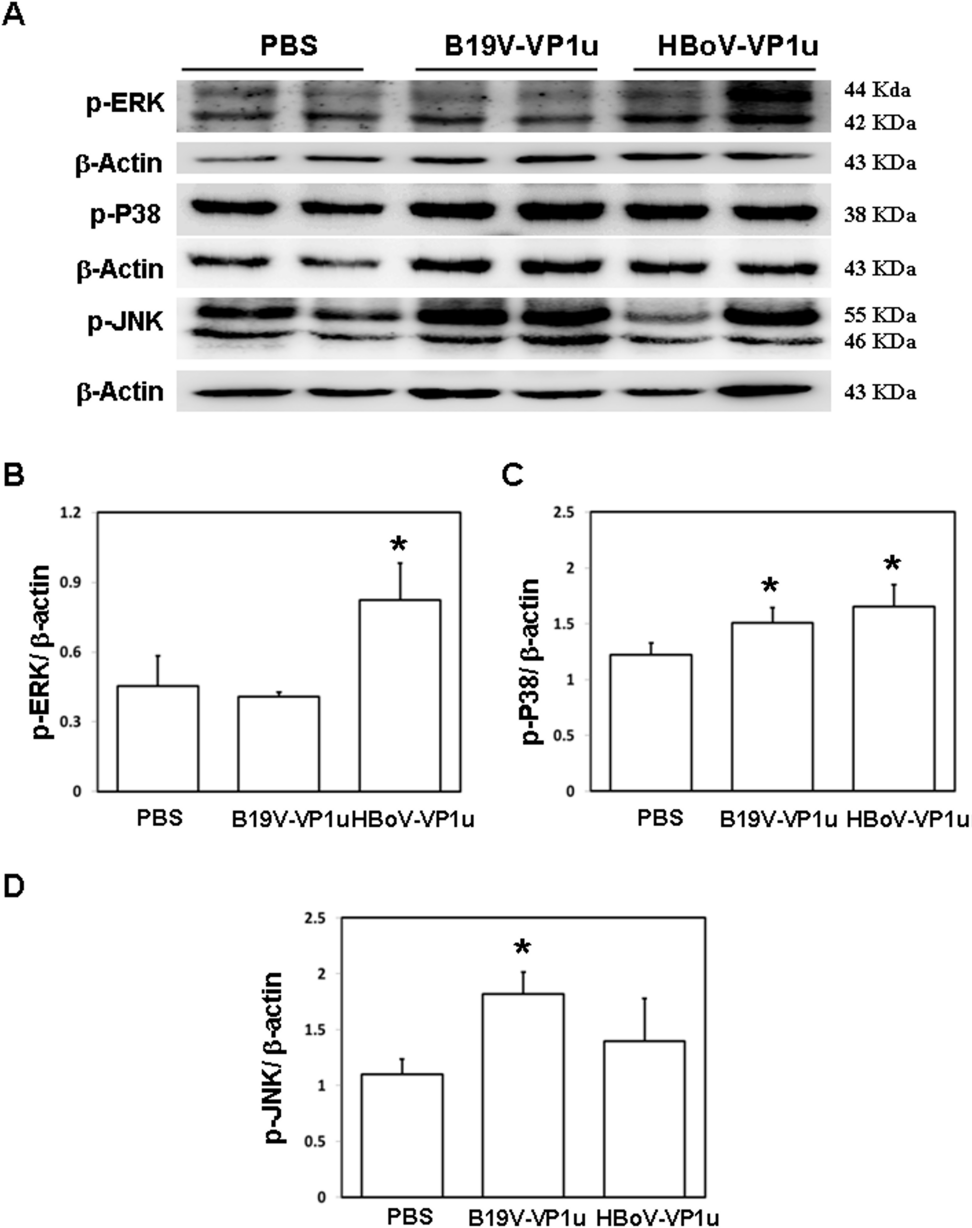
Expression of MAPK proteins in lung tissue of Balb/c mice. Expression of (A) p-ERK, p-P38 and p-JNK proteins in lung tissue of Balb/c mice that received PBS, B19V-VP1u or HBoV-VP1u protein. Bars represent the relative protein amounts of (B) p-ERK, (C) p-38, and (D) p-JNK on the basis of β-actin. Similar results were observed in three repeated experiments, and * indicates a significant difference, P<0.05.

To identify signaling molecules that may be involved in the activation of inflammatory cytokines, MMP-9 and MMP-2 in lung tissue of Balb/c mice that received B19V-VP1u or HBoV-VP1u protein, and the levels of NF-κB (p-p65), p-ERK1/2, p-p38, and p-JNK proteins were examined. Significantly increased expression of NF-κB (p-p65) protein was detected in lung tissue from Balb/c mice that received B19V-VP1u or HBoV-VP1u protein compared to that of mice that received PBS (Fig 5A). Fig 5B shows quantified results of NF-κB (p-p65) protein level relative to β-actin level. Furthermore, significantly increased expression of p-ERK1/2 proteins was detected in lung tissue from Balb/c mice that received HBoV-VP1u protein than in those of mice received PBS (Fig 6A). Significantly increased expression of p-p38 proteins was detected in lung tissue from Balb/c mice that received B19V-VP1u or HBoV-VP1u protein than in those of mice received PBS (Fig 6A). Significantly increased expression of p-JNK proteins was detected in lung tissue from Balb/c mice that received B19V-VP1u protein than in those of mice received PBS (Fig 6A). Fig 6B–6D display quantified results for levels of p-ERK1/2, p-p38 and p-JNK proteins, respectively, relative to β-actin level.

### Activity of MMP-9 and MMP-2 and expression of IL-1β and IL-6 in lung tissue of Balb/c mice received B19V-VP1uD175A or HBoV-VP1uL48R protein

**Fig 7:**
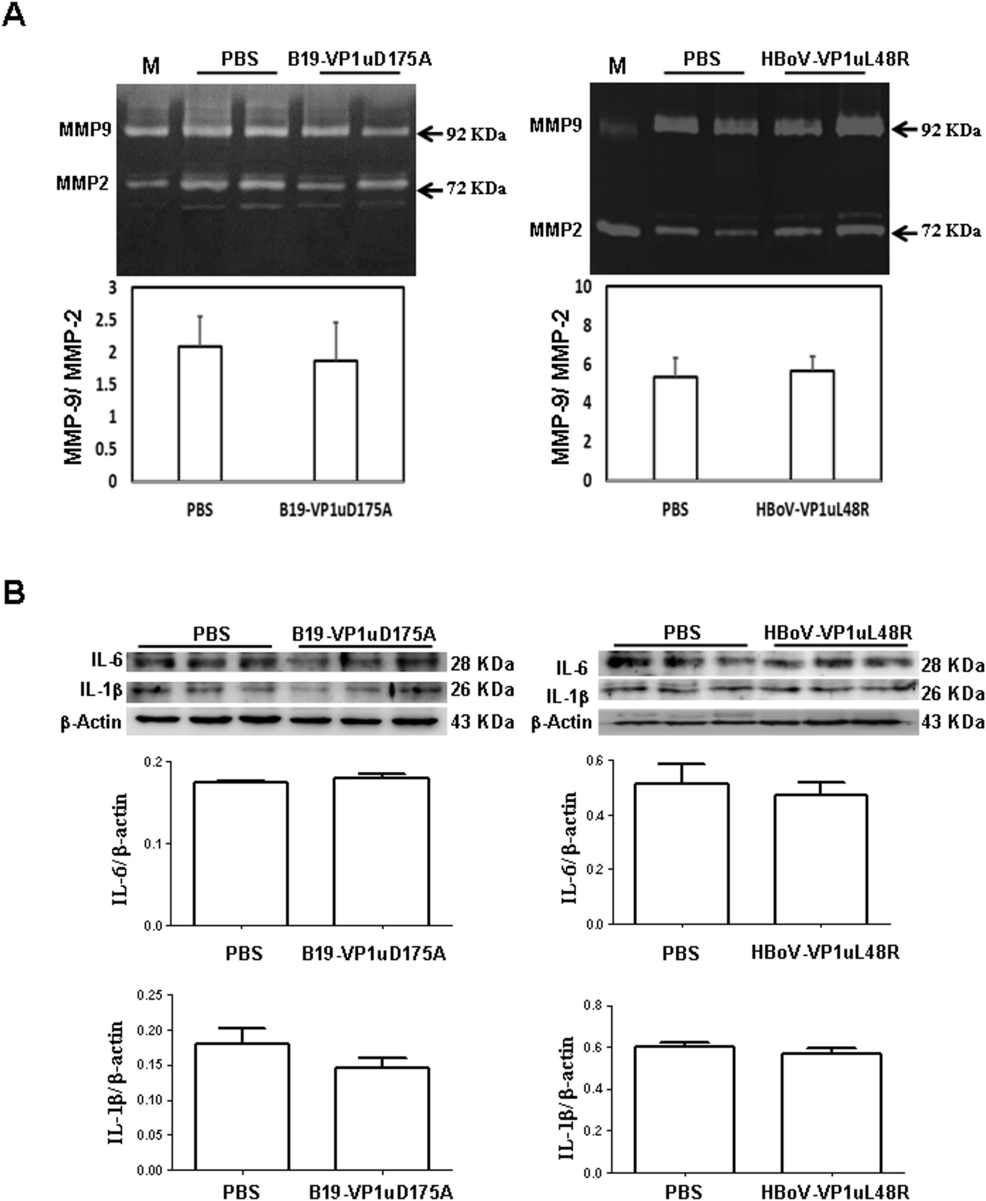
Activity of MMP-9 and MMP-2 and expression of IL-6 and IL-1β in lung tissue of Balb/c mice received mutated VP1u of B19V and HBoV. Activity of (A) MMP-9 relative and MMP-2 in lung tissues of Balb/c mice received PBS, B19-VP1uD175A or HBoV-VP1uL48R protein. Lower panels reveal the ratio of MMP-9 activity relative to MMP-2 activity. Expression of (B) IL-6 and IL-1β proteins in lung tissues of Balb/c mice received PBS, B19-VP1uD175A or HBoV-VP1uL48R protein. Bars represent the relative protein amounts of IL-6 and IL-1β on the basis of β-actin. Similar results were observed in three repeated experiments, and * indicates the significant difference, P<0.05.

To further confirm the role of sPLA2 activity within B19V-VP1u and HBoV-VP1u on lung injury, the activity of MMP-9 and MMP-2 and the expression of IL-6 and IL-1β in lung tissues of Balb/c mice received B19-VP1uD175A or HBoV-VP1uL48R protein was detected (Fig 7). No significant variation of MMP-9/MMP-2 ratio was observed in lung tissues of Balb/c mice received B19-VP1uD175A or HBoV-VP1uL48R protein as compared to those mice received PBS (Fig 7A). No significant variation was also detected in the expression of IL-6 and IL-1β proteins in lung tissues of Balb/c mice received B19-VP1uD175A or HBoV-VP1uL48R protein as compared to those mice received PBS (Fig 7B).

## Discussion

Evidence from around the world suggests a connection between human parvoviruses, including B19V and HBoV, and numerous respiratory diseases [17–19,21,26–29]. Our recent study also revealed a disruptive effect of B19V-VP1u and HBoV-VP1u on tight junctions (TJs) in A549 lung cells [24]. However, no direct evidence of the involvement of human parvoviruses in lung injury has been reported. The current study is the first to reveal that B19V-VP1u and HBoV-VP1u induce lung inflammation in naïve mice following subcutaneous injection with recombinant B19V-VP1u or HBoV-VP1u proteins, including elevated activity and protein expressions of MMP-9 and MMP-2, increased levels of inflammatory cytokines such as IL-6 and IL-1β, and lymphocyte infiltration.

The matrix metalloproteinase (MMP) family of enzymes consist of 25 endopeptidases in mice and 24 in humans, which are known to be the enzymes most responsible for proteolytic degradation of the extracellular matrix (ECM) [30–31]. Matrix metalloproteinases (MMPs) have been involved in a variety of pathophysiological processes mainly tissue destruction, fibrosis and the weakening of ECM [32]. Dysregulation of ECM composition, structure, stiffness and abundance by MMPs contributes to several pathological conditions, including atherosclerosis, fibrosis and invasive cancer [33–35]. Several MMPs, such as MMP-2 and MMP-9, are important in lung remodeling before and after birth as well as in response to environmental indices, infection, and lung injury [35]. MMP-2 and MMP-9 are elevated under stress conditions such as acute respiratory infection and allergic inflammation, and have a protective role, involving the clearance of pathogens and the production of a chemotactic gradient [35–36]. Relevant studies also indicate that increased MMP-2 and MMP-9 levels are indices of lung injury and elevated levels of inflammatory cytokines, such as IL-6 and IL-1β [37]. In this study, similar observations of significantly increased activities and levels of MMP-9 and MMP-2 proteins and of inflammatory cytokines were made in the lung tissues of naïve mice that received B19V-VP1u or HBoV-VP1u, providing evidence that B19V-VP1u and HBoV-VP1u induce lung injury.

Previously obtained evidence has demonstrated that mitogen-activated protein kinases (MAPK), such as JNK, MAPK and p38 and transcription factors, such as nuclear factor-kappaB (NF-κB) and AP-1, have a fundamental role in the differential regulation of genes for proinflammatory mediators, including MMP-9, MMP-2, IL-6 and IL-1β [38–41]. A variety of studies have demonstrated the involvement of NF-κB and MAPK in response to various growth factors, inflammatory stimuli, and pro-oxidants and that these regulate the expression of gene products that are associated with inflammation, proliferation, invasion, and angiogenesis [42–43]. Different profile of MAPK signaling, including NF-κB (p-p65), p-ERK1/2 and p-p38 protein levels, was detected in lung tissue from Balb/c mice that received B19V-VP1u or HBoV-VP1u proteins. These findings suggest that B19V-VP1u and HBoV-VP1u can differentially regulate signaling pathways for inducing pro-inflammatory mediators in lung injury.

Various types of secretory PLA2 (sPLA2) are expressed in lung tissue and can cause acute lung injury, such as diffuse alveolar damage, bronchiolitis obliterans and organizing pneumonia pattern [44–46]. Additionally, macrophages in the lung can secrete PLA2, which induces enzymes such as MMP and inflammatory cytokines such as IL-6 and IL-1β, resulting in lung inflammation [47]. The sPLA2-like activity of B19V-VP1u and HBoV-VP1u has also been linked to a wide range of pathogenic responses, including autoimmune disorders, cardiovascular disorders and respiratory infection [17,48]. Indeed, evidence has indicated that the unique region of VP1 in B19V and HBoV plays a crucial role in infection and induce inflammation in host cells or tissues due to its phospholipase A2 (PLA2)-like activity [14–16,48–50]. Although previous studies have reported that B19-VP1u protein induces myocardial inflammation and liver injury in naïve mice [48–49], little is known whether HBoV-VP1u affect the other organs in mice receiving HBoV-VP1u protein. In the current investigation, similar observations were made in naïve mice that received B19V-VP1u or HBoV-VP1u, which exhibit elevated MMP-9 and MMP-2 activity, and a markedly elevated level of inflammatory cytokines and considerable lymphocyte infiltration in lung tissues. Since the epithelia in the airway tract are vulnerable to sPLA2 owing to its proteolytic activity [51], these findings suggest the involvement of B19V-VP1u and HBoV-VP1u in inducing lung injury through sPLA2 activity. Notably, the current study further revealed that B19-VP1uD175A or HBoV-VP1uL48R has no significant effect on increasing lung MMP-9/MMP-2 ratio and expression of IL-6 and IL-1β proteins, suggesting an essential role of sPLA2 activity within B19V-VP1u and HBoV-VP1u on inducing lung injury.

## Conclusions

This study is the first to demonstrate the induction of lung injury by both B19V-VP1u and HBoV-VP1u in naïve mice by elevating MMP9/2 activation, proinflammation cytokine expression, and lymphocyte infiltration in lung tissue through differentially regulating NF-κB and MAPK signaling pathways.
